# Developing Eco-Friendly 3D-Printing Composite Filament: Utilizing Palm Midrib to Reinforce High-Density Polyethylene Matrix in Design Applications

**DOI:** 10.3390/polym16081135

**Published:** 2024-04-18

**Authors:** Ahmed El Shakhs, Noha A. Elessawy, Mohamed Fahmy El-Saka, Gasser E. Hassan, May A. Malek Ali

**Affiliations:** 1 Faculty of Architecture and Design, College of Engineering, Abu Dhabi University, Abu Dhabi 59911, United Arab Emirates; 2 Computer Based Engineering Applications Department, Informatics Research Institute, City of Scientific Research and Technological Applications (SRTA-City), New Borg El-Arab City 21934, Alexandria, Egypt; g_hassan@srtacity.sci.eg; 3 Central Laboratory for Biochemical Analysis, City of Scientific Research and Technological Applications (SRTA-City), New Borg El-Arab City 21934, Alexandria, Egypt; m_fahmy@srtacity.sci.eg; 4 Faculty of Industrial and Energy Technology, Borg Al Arab Technological University, New Borg El-Arab City 21934, Alexandria, Egypt; 5 Interior Architecture Department, Faculty of Fine Arts, Alexandria University, El-Shatby 21526, Alexandria, Egypt; may.ali@alexu.edu.eg

**Keywords:** 3D printing, filament composite, date palm frond waste, recycling, high-density polyethylene, interior design

## Abstract

Designers actively pursue the use of novel materials and concepts in furniture and interior design. By providing insights into their processing behavior and suitability for 3D-printing processes, this research helps to highlight the potential of using waste materials to create more environmentally friendly and sustainable 3D-printing filaments that can be used in furniture and interior design. Furthermore, the study evaluates the effect of incorporating palm midrib nanoparticles (DPFNPs) to reinforce a high-density polyethylene (HDPE) matrix with different loadings such as 10, 20, 30, 40, and 50 wt.%. The composites were extruded into filaments using a manual extruder, which was then utilized to fabricate 3D-printed specimens using a 3D-printing pen. The effect of adding DPFNPs on the composite’s chemical, thermal, and mechanical properties was evaluated, with a particular focus on how these modifications influence the melt flow rate (MFR) and, subsequently, the material’s printability. The results revealed that HDPE and filament composites presented similar FTIR spectra. On the other hand, the filament composites presented an increase in the thermal stability and a decrease in the mechanical strength with increasing DPFNP content in the HDPE matrix. The filaments were successfully printed using a 3D-printing pen. Thus, using DPFNPs in the HDPE matrix presents a low-cost alternative for filament production and may expand 3D-printing applications in interior and furniture design with more sustainable materials. Future work will delve into optimizing these composites for improved printability and assessing their recyclability, aiming to broaden their applications in 3D printing and beyond.

## 1. Introduction

**Figure 1:**
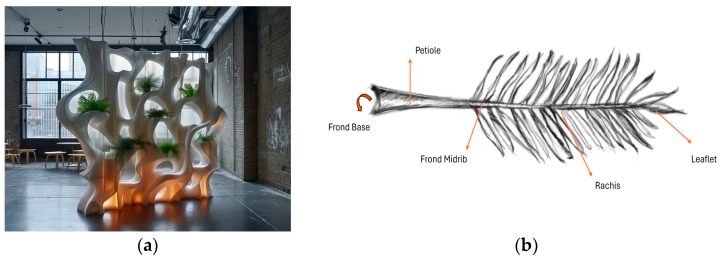
(**a**) Imaginary 3D-printed partition design generated by artificial intelligence (mid-journey Al), (**b**) date palm tree frond structure.

Incorporating eco-friendly materials into interior and furniture design is a much-sought-after objective among practitioners in the field. Designers consistently strive to explore new concepts and inventive applications, particularly within the field of furniture and product design. The rapid growth in sustainable design technologies has prompted exploration into alternative, eco-friendly materials for various applications, including 3D-printing filaments. Utilizing 3D printers in interior design has revolutionized the industry in several ways [1]: 3D printing in interior design and furniture manufacturing enhances aesthetic form, ergonomic function, and structural soundness, leading to differentiation from traditional design and material usage [2]. Multi-material magnetically assisted 3D printing offers a broader design space for manufacturing functional heterogeneous materials with exquisite microstructural features [3]. This cutting-edge technology can increase creativity and productivity in the design process, though manual work remains a component of design learning [4], and increase student/designer interest and engagement, leading to a moderate increase in novelty and a significant increase in style in a luminaire design project [5]. From bespoke furniture pieces to ornate wall panels, cladding solutions, and decorative items, 3D printing allows designers to bring their visions to life cost-effectively. One notable benefit of 3D printing is its capacity to fabricate designs with more shape complexity beyond the limitations of conventional manufacturing techniques [6]. The BANYON ECO WALL created by BigRep [7] is the pioneering creation of a fully 3D-printed green wall. It is fitted with an integrated water supply and drainage system, making it the first of its kind in the world. The complex structure of the generative design form leaves traditional technologies, such as milling or injection molding, incapable of achieving the same level of complexity and dual purpose, as shown in Figure 1a. Furthermore, the uniqueness and bespoke aspects of the products are meticulously crafted to cater to each client’s individual preferences and distinct specifications. This enhances the overall aesthetics of interior spaces and promotes a sense of exclusivity. The utilization of 3D-printing technology facilitates the advancement of sustainable design practices by mitigating waste generation through the implementation of precise manufacturing techniques, as well as the integration of novel natural materials in manufacturing filaments [8].

In conjunction with furniture and interior design, the utilization of 3D-printing technology extends to acoustic applications, facilitating the production of sound-absorbing panels and materials that augment the aural ambiance inside various environments. The adaptability of 3D printing has rendered it a significant tool for interior designers in their pursuit of novel project solutions. In addition, conventional wood joints in furniture manufacturing can be replaced with 3D-printed connectors, allowing designers to create new products with no restrictions on size, shape, or quantity of joints or color [9]. The furniture-manufacturing industry has experienced a profound integration of 3D-printing technology, which has brought about transformative changes that impact the production process, market potential, and conventional furniture industry [10]. This technology offers minor customization and diversification, allowing for diverse and form-free hardware components in furniture design [11]. Furthermore, traditional furniture manufacturers are being impacted by the rapid development of 3D-printed wood materials, including wood–plastic composite materials [10]. In street furniture manufacturing, 3D-printing technology prioritizes output size, shrinkage of material, and output angle for stability, aesthetics, and economic efficiency [12]. Three-dimensional printing can provide solutions for adjusting the height of furniture seats [13]. In addition, 3D printing significantly impacts design quality, craftsmanship, and scale in interior design, with a propensity for curved and rectilinear shapes [14]. Furthermore, 3D printing offers freedom of design, mass customization, waste minimization, and fast prototyping in various industries [15]. Prototyping has become affordable in various industries, including for manufacturing sophisticated 3D devices [16].

High-density polyethylene (HDPE) is one of the polymers that has gained prominence as a multifaceted and environmentally friendly substance [17] within the industries of interior design, furniture manufacturing, and 3D printing. HDPE, known for its exceptional strength-to-density ratio, finds extensive utility throughout a diverse range of fields, encompassing innovative furniture design, sophisticated interior embellishments, and cutting-edge 3D-printing methodologies. High-density polyethylene (HDPE) has demonstrated its superior reliability as a sustainable substitute for authentic wood. Not only does it possess a high degree of recyclability and non-toxicity, but it also presents economic benefits for both consumers and the enterprise. In the furniture industry, HDPE has been recognized for its exceptional resistance and longevity, offering an eco-friendly and economically viable alternative to traditional materials like wood. Its durability and low maintenance make it particularly suitable for outdoor furniture, where resistance to weathering is essential [18]. Furthermore, HDPE blended with lignocellulosic bagasse composite demonstrates potential in scaffolding, framework, flooring, and walls, contributing to sustainable building practices [19]. The adaptability of HDPE in various manufacturing processes, including blow molding and injection molding, allows for a wide range of applications in interior design [20]. This flexibility enables designers to explore unique forms and structures, enhancing the aesthetic and functional aspects of interior spaces. In the field of 3D printing, HDPE stands out for its improved Young’s modulus, tensile strength, and surface quality [21]. The addition of materials like carbon fiber or microcrystalline cellulose further enhances its properties, making it suitable for intricate and durable 3D-printed objects [22,23]. Environmental sustainability is another critical aspect of HDPE’s application. The biodegradation of HDPE using resources like the white rot fungus Bjerkandera adusta TBB-03 addresses environmental concerns, making it a more sustainable option [24]. Additionally, the potential for recycling HDPE in applications like pressure pipes demonstrates its role in promoting a circular economy, which is crucial in modern sustainable practices [25].

More than 100 date palm cultivars are grown for their delicious fruit and other uses. Furthermore, date palms have been essential to Arabian cultural traditions for decades. Bedouin tribes rely on date cultivation for income. Additionally, these tribes have diverse uses for the fruit peel and stone. This study presents an experimental approach to crafting a filament derived from date palm fronds (midribs), a typically underutilized agricultural byproduct. HDPE was amalgamated with shredded date palm fronds (midribs) to fabricate filaments compatible with 3D-printing processes. The fronds are structured from two sets of leaves and a midrib (petiole), as shown in Figure 1b [26].

Preliminary results highlight the potential of these filaments for creating robust, biodegradable structures with unique aesthetic appeal. The derived filament exhibited promising attributes for applications in furniture design and interior spaces, such as partitions and ceiling elements. This innovative fusion of traditional materials with contemporary techniques not only champions sustainability but also unlocks new avenues in the realm of eco-conscious interior design.

## 2. Materials and Methods

### 2.1. Fabrication of the Filament

**Figure 2:**
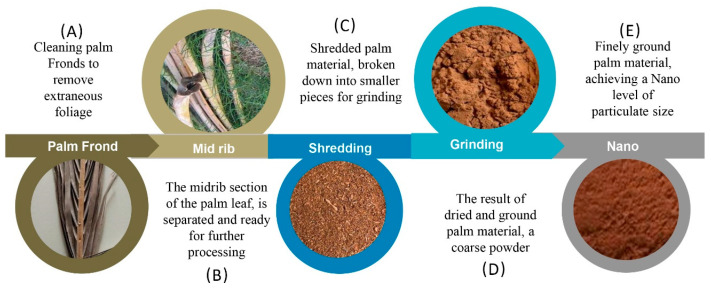
Schematic illustration of the DPFNP preparation process.

**Figure 3:**
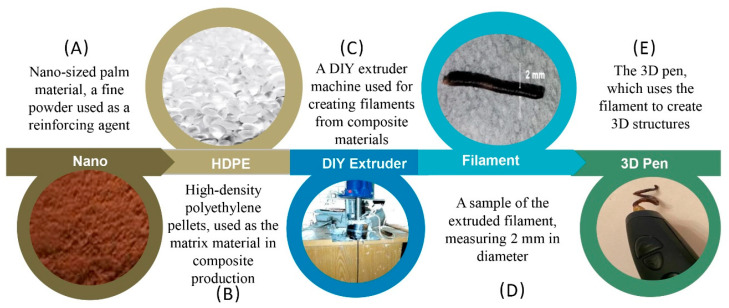
Schematic illustration of the procedure followed to prepare and test the HDPE/DPFNP composite filament.

Filament fabrication consists of two main stages: palm tree frond preparation up to nano size (Figure 2), composition mixture, and extradition (Figure 3). Figure 2 presents the process of cleaning the palm fronds and shredding the grounding up to the nano-size particles. Figure 2A,B shows the process of cleaning the palm frond of leaves, fibrillae meshes, and dust. An oven was used for the drying process, and then the shredding was only applied to part of the midrib (petiole and leaf base) (Figure 2C). Afterward, the shredded particles were ground up to a coarse powder (Figure 2D). The powder was ground up to nano size (Figure 2E).

Figure 3 presents the procedures of this study, namely, the composite preparation (Figure 3A) of the prepared nano size particles. As shown in Figure 3B, the HDPE was in the form of an industrial-grade powder with a density of 0.953 g cm^−3^, a Vicat softening temperature of 123 °C, and a melt flow rate of 26 g/10 min (SIDPEC, Cairo, Egypt), and was mixed with DPFNPs (date palm frond nano particles) in different ratios (10, 20, 30, 40, and 50 wt.%). Then, the mixture was extruded in a manual extruder at 230 °C (Figure 3C). The dispersion of the particles in the matrix is a frequent problem during the preparation of composites. Each composite was mixed in a thermomixer to achieve the optimum dispersion of the DPFNPs in the HDPE matrix. Figure 3D shows the size of the produced filament, and the filament was tested using a 3D-printing pen, as shown in Figure 3E. 

### 2.2. Characterizations

The nano-size scale of the DPFNPs was verified using Better Size Instruments Ltd., Dandong, China. Both pristine HDPE and HDPE/DPFNP composites underwent thermogravimetric analysis under a nitrogen atmosphere. The apparatus used for the measurements was SDT 650, TA Instruments, New Castle, DE, USA, with a 10 °C/min. heating rate and within a heating range of between 30 °C and 800 °C. Moreover, a DHR/20 (Discovery, Hybrid Rotational Rheometer, TA-Instruments, New Castle, DE, USA) was used to assess the melt flow rate (MFR). Fourier transform infrared (Shimadzu FTIR-8400 S-, Kyoto, Japan) surface functional group analysis was used to examine the chemical structures of the filaments. Transmittance mode was used to record data within the 4000–400 cm^−1^ range. 

**Figure 4:**
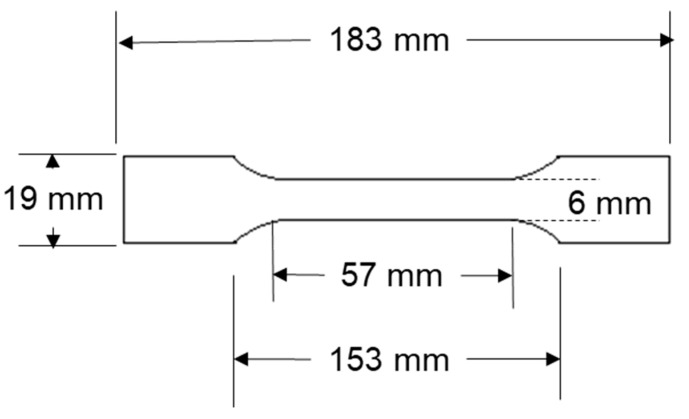
Dumbbell-shaped specimen dimensions.

Tensile tests on plastic materials are frequently conducted in accordance with the ASTM D 638-14 [27] standard to guarantee accurate and consistent results. The production method for the casting specimen is as follows: HDPE/DPFNP (high-density polyethylene/date palm frond nano particle) composite were first prepared. These composites were produced by initially processing date palm fronds to nano-size particles, mixing these particles with HDPE in varying weight percentages (10, 20, 30, 40, and 50 wt.%) mechanically, and then extruding the mixture using a manual extruder at 230 °C to a dumbbell mold to form dumbbell-shaped specimens with specific dimensions, as shown in Figure 4. Tensile tests were carried out on the corresponding specimens using a Shimadzu Autograph 5 KN, Kyoto, Japan, with standardized grips at a 5 mm/min elongation speed at room temperature. Three specimens were tested for each filament.

### 2.3. Three-Dimensional-Printing Pen

The HDPE/DPFNP filaments were printed using a 3D-printing pen to concept-proof developed filaments. The 3D-printing pen was purchased from 3Doodler pro+, Wobble Works, Inc., New York, NY, USA, with a robust dual-drive system built to run harder than any other 3D pen. The pen has a 2 mm nozzle, and the printing temperature was adjusted to 230 °C to reach the optimal melting point and a flow rate of 10 mm^3^ s^−1^.

## 3. Results

### 3.1. DPFNP Characterization

**Figure 5:**
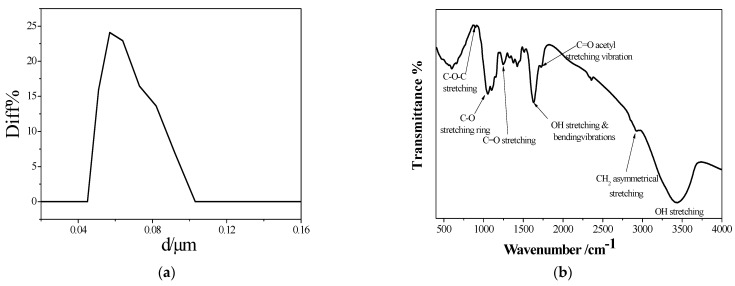
(**a**) Particle size distribution and (**b**) FTIR spectrum of DPFNPs.

The palm fronds were obtained from a local date palm plantation. After grinding, the size of the produced particles was investigated, and the nano size was confirmed from the particle size distribution (Figure 5a) using Better Size Instruments Ltd, Dandong, China. The mean particle diameter of DPFNPs ranged between 0.05 and 0.1 µm, as illustrated in Table S1 in the supplementary file.

However, the chemical composition of the dried date palm fronds was explored, and it was found that the cellulose content was about 27.26% and the hemicellulose content was about 0.67%, while the lignin content was about 8.35%. Furthermore, the FTIR analysis was used to investigate the characteristic chemical composition of DPFNPs in terms of functional groups, as shown in Figure 5b. A tiny band at 895 cm^−1^ in the FTIR spectra of DPFNP indicates the presence of C-O-C stretching, which is subject to the β-glycosidic bond of cellulose. However, the peak that showed up at 1048 cm^−1^ indicates that cellulose’s polysaccharide, the C–O stretching ring, was present. On the other hand, the C=O stretching groups of hemicellulose are represented by the peak at 1242 cm^−1^. In cellulose, the bands located at 1324 cm^−1^, 1365 cm^−1^, and 1426 cm^−1^ represent the vibrations of CH_2_ rocking, C-H deformation, and CH_2_ bending, respectively. The peaks at 1518 cm^−1^ demonstrate the presence of C=C aromatic skeletal vibration in lignin [28]. The peak at 1637 cm^−1^ is attributed to the O-H stretching and bending vibrations of the hydroxyl groups of cellulose that were hydrogen bound and the water that was absorbed, respectively. The peak located at 1732 cm^−1^ is ascribed to the acetyl of hemicellulose and lignin’s C=O stretching vibration. The CH_2_ symmetric stretching and CH_2_ asymmetric stretching groups of cellulose and hemicellulose are shown by the peaks at 2850 cm^−1^ and 2921 cm^−1^, respectively. The broad peak at 3440 cm^−1^ is associated with the hydroxyl groups’ hydrogen bond and O–H stretching vibration. In the meantime, hemicellulose and cellulose both included the bulk of oxygen functional groups [29], which positively affected the dispersed phase in the HDPE matrix.

### 3.2. Filament Characterization 

**Figure 6:**
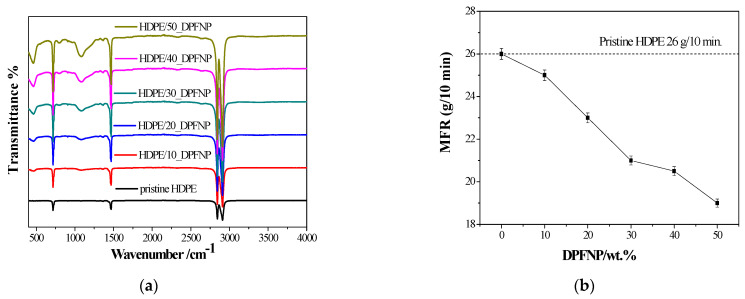
(**a**) FTIR spectra and (**b**) melt flow rate vs. DPFNP (wt.%) for pristine HDPE and HDPE/DPFNP composites.

The fabricated composite filaments with different DPFNP concentrations were named HDPE/10_DPFNP, HDPE/20_DPFNP, HDPE/30_DPFNP, HDPE/40_DPFNP, and HDPE/50_DPFNP, and their FTIR spectra are presented in Figure 6a. 

Different peaks were observed at different wavenumbers, and the intensities of these peaks varied as the DPFNP weight ratio increased from 10% to 50%. The peaks at 2842 and 2908 cm^−1^ are attributed to the C-H symmetric and asymmetric stretching vibration, respectively, which are caused by aliphatic structures [30]. Conversely, the presence of alkanes is indicated by the peak at 1468 cm^-1^, which is brought on by the C–H bond bending [31,32], and the presence of previous peaks is attributed to HDPE. Nevertheless, an extra broad peak was detected in the HDPE/DPFNP composites at a wavenumber of 1084 cm^−1^, and its intensity increased when the DPFNP weight ratio increased from 10% to 50%. The C-O stretching vibration in the DPFNP was the real cause of these peaks. Consequently, it was concluded that these functional groups were derived from DPFNPs and HDPE, suggesting a chemical and physical interaction between the two. Figure 6b shows the MFR of pristine HDPE and HDPE/DPFNP composites in grams per 10 min. It was observed that the MFR decreased as the DPFNP weight ratio increased, and the maximum MFR was identified in the case of pristine HDPE. This was anticipated, given that adding DPFNPs slows down polymer flow and makes composites more viscous. There have been previous reports of this behavior for HDPE reinforced with wood particles [33]. The fluidity rate of the composite decreased when wood particles were added to the HDPE matrix, as would be expected given their interaction with the HDPE. Additionally, the viscosity of the composite increased as a result of the wood particle addition.

**Figure 7:**
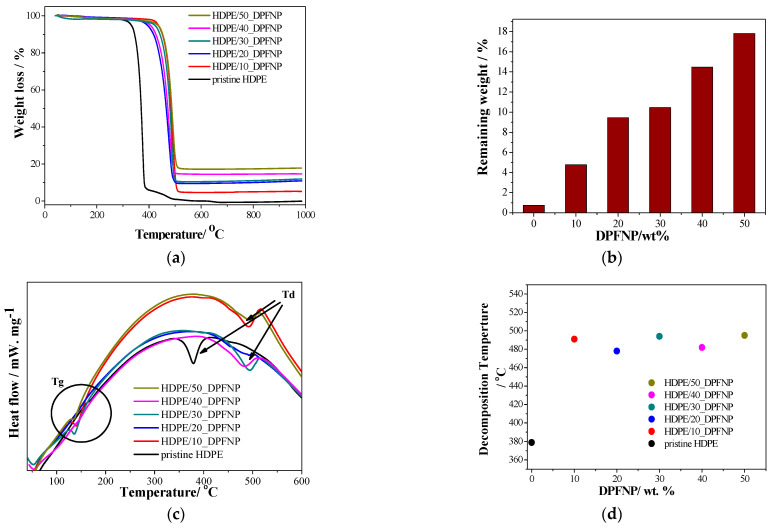
(**a**) TGA spectra, (**b**) the relation between the remaining weight % vs. DPFNP (wt.%), (**c**) DSC spectra for pristine HDPE and HDPE/DPFNP composites, and (**d**) the relation between the decomposition temperature (°C) vs. DPFNP (wt.%).

The thermal stability of pristine HDPE and HDPE/DPFNP composites between 40 °C and 1000 °C is shown in Figure 7a. The loss of HDPE mass occurred in a main single-stage degradation process that occurred in the temperature range of 300–400 °C, with another minor degradation stage occurring in the temperature range of 400–500 °C. The thermal degradation of HDPE/DPFNP composites occurred in a single step at a temperature range of between 370 °C and about 520 °C. 

Hemicellulose breaks down in the 225–325 °C range, cellulose in the 300–400 °C range [34], and lignin in a broad temperature range of between 200 and 600 °C [35]. In contrast, a little red shift in degradation temperature occurred for HDPE/DPFNP composites, and that shift was increased by increasing the DPFNP weight ratio from 10% to 50%. Thus, the thermal stability of the composites increased by increasing the filler contents [36]. The new composition provides numerous benefits in interior design. Due to its resistance to moisture, mildew, insects, and UV rays, this material is very suitable for furniture and fixtures that may be exposed to different indoor/outdoor conditions. Additionally, the utilization of HDPE/DPFNP composite in the production of plastic lumber showcases its capacity to substitute conventional wood in structural uses and outdoor shading systems, providing an eco-friendly option that withstands deterioration, harm, and the typical challenges encountered by traditional construction materials. The utilization potential of HDPE in residential building materials demonstrates its capacity for broader implementation in the design and construction industries, emphasizing its significance in the future of environmentally friendly building methods.

In Figure 7b, it was observed that the weight loss diminished as the DPFNP weight ratio increased, and the residual mass confirmed each composite’s loading of DPFNPs. As would be expected, the temperature at which the degradation happened was marginally increased by the addition of DPFNPs but not significantly. Furthermore, the temperature utilized for the 3D-printing pin was about 230 °C, which was substantially lower than the temperature at which the composites began to degrade acutely. This guaranteed that the HDPE/DPFNP filament composite did not deteriorate during the experiment, which could have impacted the obtained results.

On the other hand, Figure 7c displays the DSC graph for both pure HDPE and HDPE/DPFNP composites created at all DPFNP weight ratios. There, it is evident that the case of pristine HDPE exhibited the greatest Tg value, and the graph shows no discernible linear relationship between the DPFNP weight ratio and the Tg temperature or the differentiation of the heat flow rate (Figure S1a). However, it was also observed that the initial temperature of decomposition (Td) processes and the breakdown temperature of HDPE/DPFNP composites was increased by increasing the DPFNP weight ratio, and there was no discernible linear relationship between the DPFNP weight ratio and the Td temperature, as shown in Figure 7d and Figure S1b. This means that polymeric composites’ melting temperature is increased by incorporating cellulose nanoparticles derived from nature into the HDPE matrix [37]. 

**Figure 8:**
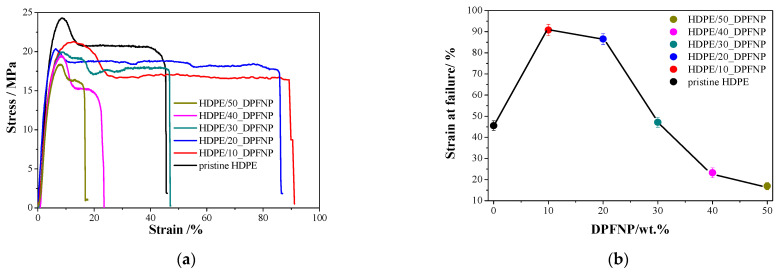
(**a**) Stress–strain behavior of pristine HDPE and HDPE/DPFNP composites obtained from the tensile test, and (**b**) the relation between strain at failure vs. DPFNP (wt.%).

Figure 8a shows the tensile stress–strain curves of pristine HDPE and HDPE/DPFNP composites containing 10–50 wt.% DPFNP. The strength and the modulus decreased linearly with the DPFNP weight fraction. However, HDPE/10_DPFNP showed the highest value of strain at failure at 50% above pristine HDPE, and then the values of strain at failure decreased by increasing the DPFNP content, as shown in Figure 8b. Furthermore, the maximal stress and material stiffness decreased with increasing DPFNP content, which means that the molecules were less mobile, which consequently decreased the strain. The available stress transfer area decreased with increasing DPFNP loading, which was the cause of the decrease in tensile stress and modules. As a result, the decrease in stress transfer area at a high DPFNP weight percentage made the nano-composite more brittle and reduced the composite’s tensile strength. There have been prior reports of this tensile modulus behavior for HDPE reinforced with short hemp fiber [38]. The obtained results will be taken into consideration when determining the optimal floating design elements for furniture and product design utilizing 3D printing. Additionally, these results will serve as criteria when selecting forms that are generated by generative design applications.

### 3.3. Three-Dimensional-Printing Pen

**Figure 9:**
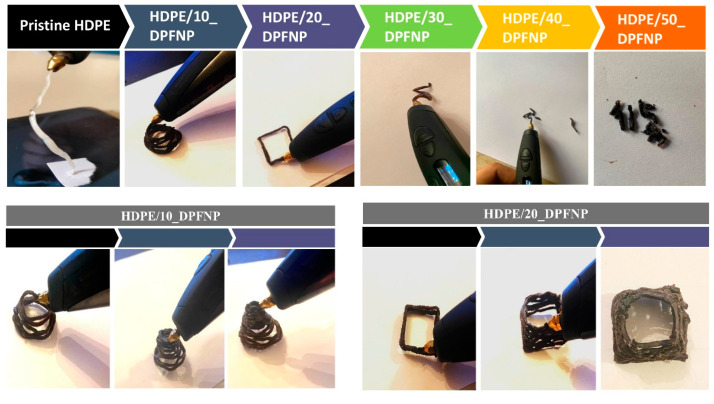
Three-dimensional-printing pen samples of HDPE/DPFNP filament composites.

Tests were conducted on filament composites using a 3D-printing pen. All of the 3D-pen-printed filaments are displayed in Figure 9, which demonstrates that the HDPE/10_DPFNP filament composite outperformed the HDPE/20_DPFNP filament composite in terms of shape, appearance, and stability, as shown in Figure S1 in the supplementary information. However, printing with a pen became more challenging as the filament began to disintegrate when the DPFNP percentage was increased to 50%. Even though the behavior of the HDPE/10_DPFNP filament composite and pristine HDPE behaved similarly when printed with a 3D pen, it is essential to emphasize the environmental commitment made by providing a new use for natural fiber waste and creating the opportunity to create a new product with better mechanical qualities and less environmental impact. Thus, HDPE/10_DPFNP filament composite presents as a promising material for application in furniture manufacture and interior design.

Comparing the newly developed date palm midrib nanoparticle-reinforced HDPE (HDPE/DPFNP) composites for 3D-printing filament to similar composites in recent research reveals several advancements and unique properties, such as:

- Mechanical performance and impact resistance were the main topics of Caminero et al.’s investigation into the impact performance of continuous fiber-reinforced thermoplastic composites (CFRTPCs) made utilizing FDM. They discovered that the impact strength of these composites increased with the amount of fiber, with samples reinforced with glass fiber showing the highest impact strength. In comparison to traditional materials, this work highlights how fiber reinforcements can improve the mechanical and impact properties of thermoplastic composites [39].
- Printing quality and mechanical properties with nanocellulose: HDPE and chemically altered cellulose nanofibrils were combined to create a bio-based filament by Dalloul et al. [40]. Because the modified cellulose nanofibrils were fillers, the mechanical characteristics and print quality of this composite improved. This demonstrates how natural fillers, including palm midrib, can be used to improve HDPE composites and increase their characteristics for 3D printing.
- Sustainability and environmental impact: With the growing need for environmentally friendly materials in additive manufacturing, attention is being paid to the use of sustainable and renewable fillers, like cellulose nanofibrils and palm midrib. By using bio-based fillers, these composites not only have enhanced qualities but also support environmental sustainability.
- Enhanced printability with modified fibers: In contrast, modified cellulose nanofibrils were used to address the significant shrinkage and warping issues with HDPE in 3D printing [40]. Better printability and mechanical qualities result from this alteration, which also improves compatibility and dispersion within the HDPE matrix. This method emphasis the benefit of palm midrib in enhancing the characteristics and printability of HDPE composites.
- Comparison with other reinforcements: Comparing CFRTPCs and nanocellulose-based filaments with other reinforcements has shown the advantages of both fiber reinforcement and natural fillers [41,42]. However, HDPE/DPFNP composites offer a new material choice that combines the benefits of bio-based reinforcement with the well-known properties of HDPE. In terms of mechanical characteristics, sustainability, and prospective applications in 3D printing, this comparison is intriguing.

In summary, these studies show that the creation of HDPE composites reinforced with date palm midrib nanoparticles advances the growing field of environmentally friendly, high-performing materials for 3D printing. These composites, which are in line with the objectives of sustainability and material innovation in additive manufacturing, present a viable substitute for conventional and other bio-based composites by utilizing distinctive natural resources like palm midrib.

## 4. Conclusions

In this study, DPFNPs were used as a naturally reinforced material for HDPE in five different concentrations ranging between 10 wt.% and 50 wt.% to produce eco-friendly and cost-effective filaments to be examined through a variety of tests and compared with the performance of pristine HDPE using a 3D-printing pen. The obtained composites showed higher thermal stability and a lower melting flow index than pristine HDPE upon increasing the DPFNP weight ratio from 10 wt.% to 50 wt.%. On the other hand, by increasing the DPFNP weight ratio, the composite’s tensile strength increased and the composite’s stiffness decreased. When printed with a 3D-printer pen, the HDPE/10_DPFNP filament composite and virgin HDPE exhibited comparable behaviors. In summary, the diverse applications of HDPE composites with natural waste materials in furniture, interior design, and 3D printing highlight their adaptability, durability, and potential for sustainability. The composite’s ability to be molded into various shapes and enhanced with eco-friendly additives makes it a valuable material in the creation of innovative and environmentally friendly design solutions. 

## Data Availability

Data are contained within the article.

## Supplementary Materials

The following supporting information can be downloaded at: https://www.mdpi.com/article/10.3390/polym16081135/s1, Figure S1: DSC curve for pristine HDPE and HDPE/DPFNP composites at different temperature ranges: (a) at a temperature range of between 40 and 200 °C and (b) at a temperature range of between 300 and 600 °C; Table S1: Particle size distribution table.
