# Supplementary material for: Developing Eco-Friendly 3D-Printing Composite Filament: Utilizing Palm Midrib to Reinforce High-Density Polyethylene Matrix in Design Applications
Source: Polymers (Basel). 2024 Apr 18;16(8):1135. doi: 10.3390/polym16081135 (PMC11054328; doi:10.3390/polym16081135)
Supplement: Supplementary file 1 [file polymers-16-01135-s001.zip › polymers-2956851-supplementary.pdf]

Developing Eco-Friendly 3D-Printing Composite Filament: Utilizing Palm Midrib to Reinforce High-Density Polyethylene Matrix in Design Applications

Ahmed El Shakhs [1,*], Noha A. Elessawy [2,*], Mohamed Fahmy El-Saka [3], Gasser E. Hassan [2,4] and May A. Malek Ali [5]

1 Faculty of Architecture and Design, College of Engineering, Abu Dhabi University, Abu Dhabi 59911, United Arab Emirates
2 Computer Based Engineering Applications Department, Informatics Research Institute, City of Scientific Research and Technological Applications (SRTA-City), New Borg El-Arab City 21934, Alexandria, Egypt; g_hassan@srtacity.sci.eg
3 Central Laboratory for Biochemical Analysis, City of Scientific Research and Technological Applications (SRTA-City), New Borg El-Arab City 21934, Alexandria, Egypt; m_fahmy@srtacity.sci.eg
4 Faculty of Industrial and Energy Technology, Borg Al Arab Technological University, New Borg El-Arab City 21934, Alexandria, Egypt
5 Interior Architecture Department, Faculty of Fine Arts, Alexandria University, El-Shatby 21526, Alexandria, Egypt; may.ali@alexu.edu.eg
* Correspondence: ahmed.elshakhs@adu.ac.ae (A.E.S.); nony_essawy@yahoo.com (N.A.E.)

Table S1: Particle size distribution table

Diam/µm	Diff%	Cum%	Diam/µm	Diff%	Cum%	Diam/µm	Diff%	Cum%	Diam/µm	Diff%	Cum%
0.020-0.022	0.00	0	0.379-0.427	0.00	100	7.211-8.112	0.00	100	136.9-154.0	0.00	100
0.022-0.025	0.00	0	0.427-0.480	0.00	100	8.112-9.126	0.00	100	154.0-173.2	0.00	100
0.025-0.028	0.00	0	0.480-0.540	0.00	100	9.126-10.26	0.00	100	173.2-194.9	0.00	100
0.028-0.032	0.00	0	0.540-0.608	0.00	100	10.26-11.54	0.00	100	194.9-219.3	0.00	100
0.032-0.036	0.00	0	0.608-0.684	0.00	100	11.54-12.99	0.00	100	219.3-246.7	0.00	100
0.036-0.040	0.00	0	0.684-0.769	0.00	100	12.99-14.61	0.00	100	246.7-277.5	0.00	100
0.040-0.045	0.00	0	0.769-0.865	0.00	100	14.61-16.44	0.00	100	277.5-312.2	0.00	100
0.045-0.051	0.00	0	0.865-0.974	0.00	100	16.44-18.48	0.00	100	312.2-351.2	0.00	100
0.051-0.057	15.93	15.93	0.974-1.095	0.00	100	18.48-20.80	0.00	100	351.2-395.1	0.00	100
0.057-0.064	24.08	40.01	1.095-1.232	0.00	100	20.80-23.40	0.00	100	395.1-444.5	0.00	100
0.064-0.073	22.92	62.93	1.232-1.386	0.00	100	23.40-26.33	0.00	100	444.5-500.0	0.00	100
0.073-0.082	16.46	79.39	1.386-1.560	0.00	100	26.33-29.62	0.00	100	500.0-562.5	0.00	100
0.082-0.092	13.64	93.03	1.560-1.755	0.00	100	29.62-33.32	0.00	100	562.5-632.8	0.00	100
0.092-0.103	6.97	100	1.755-1.974	0.00	100	33.32-37.49	0.00	100	632.8-711.9	0.00	100
0.103-0.116	0.00	100	1.974-2.221	0.00	100	37.49-42.17	0.00	100	711.9-800.9	0.00	100
0.116-0.131	0.00	100	2.221-2.498	0.00	100	42.17-47.44	0.00	100	800.9-900.9	0.00	100
0.131-0.148	0.00	100	2.498-2.811	0.00	100	47.44-53.37	0.00	100	900.9-1013	0.00	100
0.148-0.166	0.00	100	2.811-3.162	0.00	100	53.37-60.04	0.00	100	1013-1140	0.00	100
0.166-0.187	0.00	100	3.162-3.557	0.00	100	60.04-67.55	0.00	100	1140-1282	0.00	100
0.187-0.210	0.00	100	3.557-4.002	0.00	100	67.55-75.98	0.00	100	1282-1443	0.00	100
0.210-0.237	0.00	100	4.002-4.502	0.00	100	75.98-85.49	0.00	100	1443-1623	0.00	100
0.237-0.266	0.00	100	4.502-5.065	0.00	100	85.49-96.17	0.00	100	1623-1826	0.00	100
0.266-0.300	0.00	100	5.065-5.698	0.00	100	96.17-108.1	0.00	100	1826-2054	0.00	100
0.300-0.337	0.00	100	5.698-6.410	0.00	100	108.1-121.7	0.00	100	2054-2311	0.00	100
0.337-0.379	0.00	100	6.410-7.211	0.00	100	121.7-136.9	0.00	100	2311-2600	0.00	100

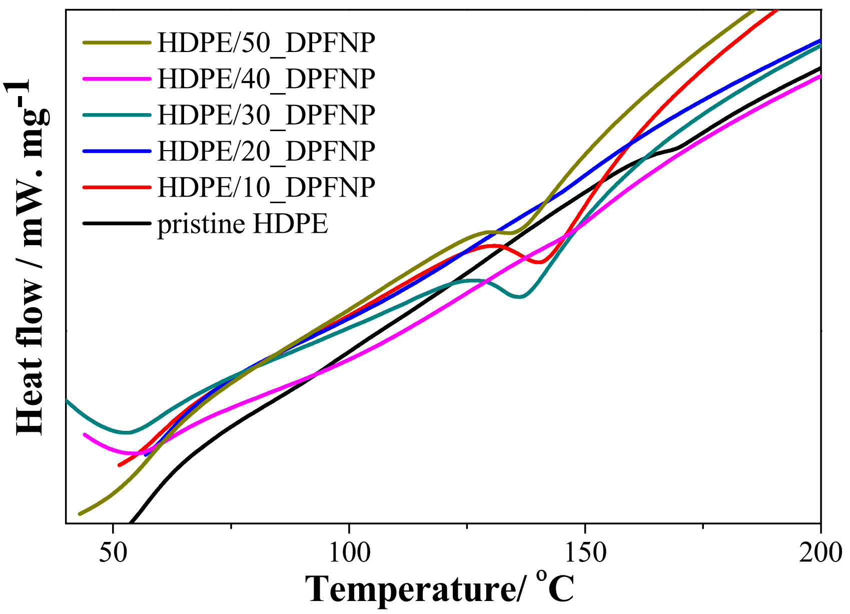

(a)

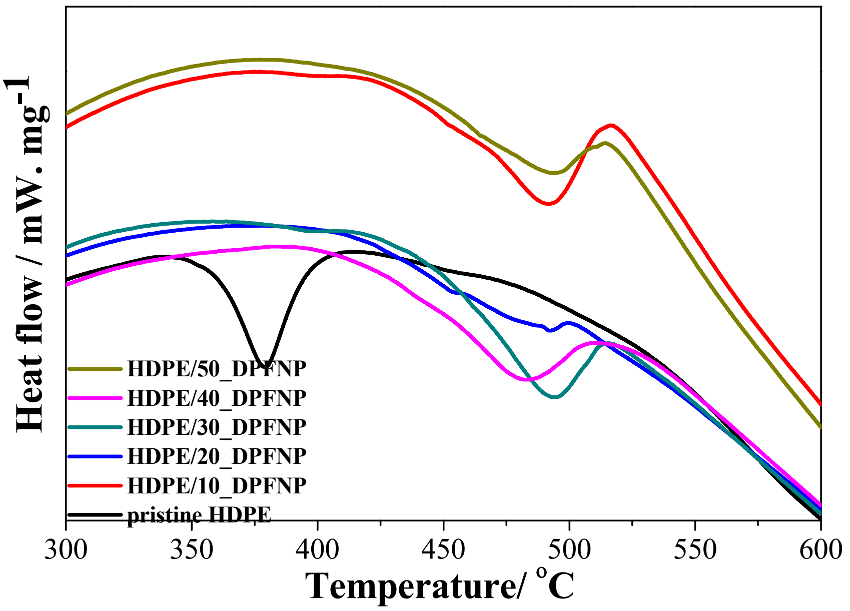

(b)

Figure S1: DSC curve for pristine HDPE and HDPE/DPFNP composites at different temperature range (a) at temperature range between 40-200°C and (b) at temperature range between 300-600°C.
